# Exposure to per- and polyfluoroalkyl substances is associated with impaired cardiovascular health: a cross-sectional study

**DOI:** 10.3389/fpubh.2024.1418134

**Published:** 2024-08-29

**Authors:** Shuli Zong, Lin Wang, Sutong Wang, Yongcheng Wang, Yuehua Jiang, Liping Sun, Yingying Zong, Xiao Li

**Affiliations:** ^1^First Clinical Medical College, Shandong University of Traditional Chinese Medicine, Jinan, China; ^2^Department of Cardiovascular Diseases, Shandong University of Traditional Chinese Medicine Affiliated Hospital, Jinan, China; ^3^Central Laboratory, Shandong University of Traditional Chinese Medicine Affiliated Hospital, Jinan, China; ^4^Department of Endocrine Tumor Intervention, Central Hospital Affiliated to Shandong First Medical University, Jinan, China; ^5^Department of Business Administration, Shandong Yingcai University, Jinan, China

**Keywords:** cardiovascular health, perfluoroalkyl and polyfluoroalkyl substances, NHANES, Life’s essential 8, cross-sectional study

## Abstract

**Background:**

Per- and polyfluoroalkyl substance (PFAS) exposure and cardiovascular disease are controversial. We aimed to assess the association between serum PFAS exposure and cardiovascular health (CVH) in U.S. adults.

**Methods:**

We analyzed serum PFAS concentration data of U.S. adults reported in the National Health and Nutrition Examination Survey (NHANES) study (2005–2018). We employed two weighted logistic regression models and a restricted cubic spline (RCS) to examine the association between each PFAS and impaired CVH (defined as moderate and low CVH). Quantile g-computation (Qgcomp) and weighted quantile sum (WQS) analysis were used to estimate the effects of mixed exposures to PFASs on impaired CVH.

**Results:**

PFAS were associated with an increased risk of impaired CVH (OR_PFNA_: 1.40, 95% CI: 1.09, 1.80; OR_PFOA_: 1.44, 95% CI: 1.10, 1.88; OR_PFOS_: 1.62, 95% CI: 1.25, 2.11). PFOA and PFOS exhibited nonlinear relationships with impaired CVH. Significant interactions were observed for impaired CVH between race/ethnicity and PFHxS (*p* = 0.02), marital status and PFOA (*p* = 0.03), and both marital status and race/ethnicity with PFOS (*p* = 0.01 and p = 0.02, respectively). Analysis via WQS and Qgcomp revealed that the mixture of PFAS was positively associated with an increased risk of impaired CVH.

**Conclusion:**

PFNA, PFOA, and PFOS exposure are associated with an increased risk of impaired CVH in U.S. adults. Race/ethnicity and marital status may influence CVH. Reducing PFAS exposure could alleviate the burden of disease associated with impaired CVH.

## 1 Introduction

Cardiovascular disease (CVD) constitutes a significant global health burden, exhibiting a rising incidence and a trend towards affecting younger individuals (1). The 2024 US Heart Disease and Stroke Statistics Report revealed that approximately 127.9 million Americans aged ≥20 years (48.6%) are afflicted by CVD, resulting in an average annual cost of 42.23 billion dollars (2). The American Heart Association (AHA) reported that a substantial number of CVDs stem from health and behavioral factors. To address this, they developed Life’s Essential 8 (LE8), which emphasizes eight critical aspects of cardiovascular health (CVH). These include four health behaviors [diet, physical activity (PA), tobacco/nicotine exposure, and sleep health] and four health factors [body mass index (BMI), lipids, blood glucose, and blood pressure (BP)] (3). Research indicates that maintaining high CVH is linked with reducing the risk of multiple health risks (4, 5). Despite implementing various public health and healthcare policies and programs in the U.S. aimed at preventing CVD, the overall CVH scores for U.S. adults have remained largely unchanged over the past decade (6).

In parallel with ongoing efforts to manage CVD, emerging concerns about environmental contaminants, such as per—and polyfluoroalkyl substances (PFASs), have gained attention. PFASs are synthetic compounds that are found in humans and animals worldwide. Characterized by their distinctive chemical structure and properties, PFASs are commonly used in various industries, such as surfactants, food packaging, waterproof coatings, and nonstick coatings on cookware (7). The half-life of long-chain PFASs can range from 3 years to decades, so even a single serum measurement can represent long-term exposure to PFASs (8, 9). Owing to their resistance to biodegradation, PFAS tend to accumulate in the biosphere through the food chain. This results in persistent health effects, including endocrine disruption, immune-inflammatory responses, and cytotoxicity (10–12). Previous studies linked PFAS exposure to an array of CVDs, and a cross-sectional study in the U.S. revealed that the risk of total CVD increased with increasing levels of PFAS, with the highest quartile of PFAS levels increasing the risk of CVD by 45% compared with the lowest quartile (13). Furthermore, another study showed that higher levels of perfluorooctanesulfonic acid (PFOS) and perfluorononanoic acid (PFNA) were associated with an increased risk of stroke in U.S. adults, in addition to an increased risk of coronary heart disease with elevated PFNA levels (14). PFASs have also been associated with cardiovascular risk factors such as insulin resistance, metabolic syndrome, and dyslipidemia, and in these high-risk populations, PFAS exposure may exacerbate adverse cardiovascular events (12, 15, 16). A prospective study of 666 pre-diabetic adults revealed that increased PFAS exposure was associated with an increased risk of coronary and thoracic aortic calcification (17). However, these studies focused primarily on the prevalence of CVD rather than CVH status. Recent evidence has challenged the traditional view. Two independent Swedish population-based cohort studies reported no statistically significant associations between PFAS levels and CVD incidence, and a meta-analysis of results from five independent cohort studies revealed a modest inverse association between perfluorooctanoic acid (PFOA) levels and CVD incidence (18). These conflicting findings suggest a multifaceted and complex relationship between PFAS exposure and CVD. Thus, exploring the direct link between PFASs and CVH may lead to a deeper understanding of the systemic effects of PFASs, thereby enabling the development of more effective preventive and intervention strategies.

In this study, we used the LE8 score to assess CVH and a cross-sectional approach to examine the relationship between PFAS exposure and CVH in U.S. adults based on nationally representative National Health and Nutrition Examination Survey (NHANES) data. Additionally, we analyzed the associations between PFAS exposure and CVH in different subpopulations.

## 2 Methods

### 2.1 Study design and population

The NHANES is conducted by the National Center for Health Statistics (NCHS) in the U.S. and is a biennial survey of representative data on demographics, socioeconomic status, health, and nutrition using a stratified multistage probability sampling design. The NHANES datasets are publicly available on the official website. This study used data from seven NHANES cycles from 2005 to 2018 in a cross-sectional design. A total of 15,868 participants were subjected to PFAS measurements over the seven survey cycles. We first excluded 3,461 adolescents under 20 to focus on the adult population. We subsequently excluded another 3,671 participants who lacked complete LE8 data. Furthermore, we excluded participants with incomplete covariate information (*n* = 1,371) and those lacking complete PFAS data (*n* = 181), resulting in a final analysis of 7,184 participants (Supplementary Figure S1). The study was approved by the NCHS Institutional Review Board, and all participants provided written informed consent. It was also conducted under the Strengthening the Reporting of Observational Studies in Epidemiology (STROBE) guidelines (Supplementary Table S6) (19).

### 2.2 Measurement of serum PFAS exposure

Our study focused on four PFAS substances, perfluorohexane sulfonic acid (PFHxS), PFOS, PFOA, and PFNA, as their detection rates exceeded 90% in all analyzed samples from the NHANES (2005–2018). According to the NHANES data description document, in the 2005–2012 cycles, NHANES directly quantified these four PFAS, whereas in the 2013–2018 cycles, a quantitative analysis of four different structural isomers of PFOS and PFOA was performed. The NHANES survey analysis guidelines suggest that the total PFOS concentration includes linear PFOS (n-PFOS) and the monomethyl branched isomers of PFOS (Sm-PFOS), whereas the total PFOA concentration is derived by combining linear PFOA (n-PFOA) and the branched isomer of PFOA (Sb-PFOA). Samples falling below the limit of detection were recorded as the limit of detection divided by the square root of 2 (20).

### 2.3 Assessment of CVH

The LE8 score was used to assess CVH and consisted of two main components: four health behaviors and four health factors. For diet scores, the Healthy Eating Index 2015 (HEI-2015) was calculated from 24-h dietary recall data collected through interviews and phone follow-ups and analyzed with the United States Department of Agriculture’s Food Patterns Equivalents Database. The total reported weekly duration of moderate or greater activities was used for the PA scores. For nicotine exposure scores, questionnaires assessed the use of combustible cigarettes, e-cigarettes, other tobacco products, and household secondhand smoke exposure. For sleep health scores, self-reported sleep duration was used. For health factors, BMI scores were calculated from weight and height measurements taken by trained researchers at the Mobile Examination Center (MEC). For BP scores, measurements were taken by trained personnel on the MEC, with additional data on antihypertensive medication use obtained from questionnaires. For lipid scores, blood samples collected from the MEC were analysed in a central laboratory, where non-HDL cholesterol was calculated by subtracting HDL cholesterol from total cholesterol, with information on lipid-lowering medication use gathered from questionnaires. For blood glucose scores, fasting blood samples were tested for fasting glucose or HbA1c in a central laboratory, and data on diabetes history and glucose-lowering medication use were obtained via questionnaires. Each component was rigorously scored from 0 to 100 according to AHA guidelines, and the total LE8 score was computed as the unweighted average of these scores. Based on the total score, CVH was categorized as low (0–49), moderate (50–79), or high (80–100). Impaired CVH was defined as moderate and low levels of CVH. Details of the LE8 score were shown in Supplementary Table S1.

### 2.4 Assessment of covariates

Information on demographic and health-related factors, including age, sex, race/ethnicity, education level, marital status, poverty income ratio (PIR), health insurance, medical history, and alcohol consumption, was collected via standardized questionnaires. In detail, race/ethnicity was divided into non-Hispanic whites, non-Hispanic blacks, Mexican Americans, and others. Marital status was divided into coupled (defined as married or living with a partner) and single or separated. Education level was divided into three categories: high school or less, some college or associate degree, and college graduate degree or above. Health insurance status was recorded as yes or no (21). Alcohol consumption was obtained via 24-h dietary recall and categorized according to intake (22). Depression was assessed via the PHQ9 scale, with a score of 10 or higher considered indicative of depression. Diabetes diagnosis was based on medical and medication history and blood glucose levels. The glomerular filtration rate (eGFR) was estimated via the CKD-EPI Formula (23). The urinary albumin-to-creatinine ratio (uACR) and the use of antihypertensive and antidiabetic medications were also included in the analysis.

### 2.5 Statistical methods

To ensure the national representativeness of the research, we followed NHANES guidelines and considered the complex sampling design of the survey. Continuous variables that followed a normal distribution were compared between groups via *t*-tests and are expressed as the means and standard errors. Non-normally distributed continuous variables were compared via Wilcoxon tests and are expressed as medians (interquartile ranges). Categorical variables were analyzed via chi-square tests, and the data are presented as frequencies and weighted percentages (%).

We employed weighted multifactor logistic regression to investigate the correlation of each PFAS with impaired CVH. To improve the model fit, we log-transformed the concentrations of each PFAS (24). We also included the PFAS quartiles in the analysis as categorical variables, with the Q1 group (low) as the control, and calculated odds ratios (ORs) and their corresponding 95% confidence intervals (95% CIs). Two different statistical models were used: the crude model without adjustment for any variables and Model 1, which accounted for age, sex, race/ethnicity, education level, marital status, PIR, alcohol consumption, health insurance, diabetes, depression, antihypertensive or lipid-lowering medication, CVD, eGFR, and the uACR.

We implemented RCS regression model, adjusting for the covariates described in Model 1 to further investigate the potential relationship between PFASs and impaired CVH. To ensure the representativeness of the results for the U.S. population, RCS modelling was performed with NHANES sample weights. The number and placement of knots in the RCS model were determined based on the Akaike information criterion (AIC) to achieve an optimal balance between model fit and avoiding overfitting. In constructing the RCS model for PFHxS and PFNA, three knots were positioned at the 10th, 50th, and 90th percentiles of their respective distributions, with the medians used as reference points. In the RCS model for PFOA and PFOS, four knots were placed at the 5th, 35th, 65th, and 95th percentiles of their distributions, with the inflexion points serving as reference points. Moreover, subgroup analyses of age, sex, race/ethnicity, PIR, education level, marital status, and health insurance status were performed to identify potential subgroups and to perform interaction tests.

Additionally, we used quantile g-computation (Qgcomp) and weighted quantile sum (WQS) analysis to test the association of mixed PFAS exposure with impaired CVH (25). To increase the reliability of our findings, we performed a sensitivity analysis and re-evaluated the associations via weighted multifactorial logistic regression after excluding participants with CVD, diabetes, or depression.

All the statistical procedures were conducted via version 4.3.2 of the R software. All the statistical tests were two-sided, with *p* values less than 0.05 indicating statistical significance.

## 3 Results

### 3.1 Population characteristics

The participants were categorized into groups with high CVH and impaired CVH. As shown in Table 1, the study included 7,184 participants, with a mean age of 47.97 years. Female participants outnumbered male participants (51.27% vs. 48.73%). The average LE8 score was 68.39, with most participants being non-Hispanic white, possessing high school or less, being coupled, enjoying good economic status, being mild drinkers, and having health insurance. Overall, 12.05% of the participants had diabetes, 8.03% had CVD, and 32.89% were prescribed medication for hypertension or lowering lipids. A total of 5,802 individuals exhibited impaired CVH. Compared with those in the high-CVH group, those with impaired CVH tended to be older, male, non-Hispanic black, with high school education or less, single or separated, economically disadvantaged, former drinkers, no health insurance, diabetic, depressed, and on antihypertensive or lipid-lowering medication. Additionally, these participants also presented lower eGFRs and higher uACR values.

**Table 1:** Baseline characteristics of the study population according to high CVH and impaired CVH.

Variable	Total (*n* = 7,184)	High CVH (*n* = 1,382)	Impaired CVH (*n* = 5,802)	*p*-value
Age (years)	47.97 ± 0.33	42.47 ± 0.56	49.63 ± 0.34	< 0.0001
**Age group (*n*, %)**				< 0.0001
20–39	2,282 (33.84)	709 (49.38)	1,573 (29.14)	
40–59	2,512 (40.29)	409 (34.80)	2,103 (41.95)	
> = 60	2,390 (25.87)	264 (15.82)	2,126 (28.91)	
**Gender (*n*, %)**				< 0.0001
Female	3,672 (51.27)	833 (59.72)	2,839 (48.72)	
Male	3,512 (48.73)	549 (40.28)	2,963 (51.28)	
**Race/ethnicity (*n*, %)**				< 0.0001
Non-Hispanic Black	1,457 (9.80)	161 (5.06)	1,296 (11.24)	
Non-Hispanic white	3,397 (71.55)	707 (75.29)	2,690 (70.42)	
Mexican American	1,029 (7.32)	180 (6.63)	849 (7.53)	
Other	1,301 (11.32)	334 (13.02)	967 (10.81)	
**Education level (*n*, %)**				< 0.0001
High school or less	3,154 (35.84)	333 (17.19)	2,821 (41.48)	
Some college or associate degree	2,185 (31.42)	389 (26.73)	1,796 (32.84)	
College graduate or above	1,845 (32.74)	660 (56.08)	1,185 (25.68)	
**Marital status (*n*, %)**				0.03
Coupled	4,430 (65.75)	899 (69.07)	3,531 (64.74)	
Single or separated	2,754 (34.25)	483 (30.93)	2,271 (35.26)	
**PIR (*n*, %)**				< 0.0001
<1.3	2,061 (18.47)	276 (12.29)	1,785 (20.34)	
1.3–3.5	2,692 (35.13)	464 (29.26)	2,228 (36.91)	
>3.5	2,431 (46.40)	642 (58.45)	1,789 (42.75)	
**Alcohol consumption status (*n*, %)**				< 0.0001
Never	912 (9.63)	213 (11.38)	699 (9.10)	
Former	1,207 (13.71)	125 (7.50)	1,082 (15.59)	
Mild	2,562 (38.81)	549 (42.77)	2,013 (37.61)	
Moderate	1,132 (17.91)	264 (21.79)	868 (16.74)	
Heavy	1,371 (19.93)	231 (16.56)	1,140 (20.96)	
**Health insurance (*n*, %)**				< 0.001
No	1,386 (15.17)	237 (11.84)	1,149 (16.18)	
Yes	5,798 (84.83)	1,145 (88.16)	4,653 (83.82)	
**Diabetes (*n*, %)**				< 0.0001
No	6,050 (87.95)	1,352 (98.00)	4,698 (84.92)	
Yes	1,134 (12.05)	30 (2.00)	1,104 (15.08)	
**Depression (*n*, %)**				< 0.0001
No	6,577 (92.96)	1,339 (97.64)	5,238 (91.55)	
Yes	607 (7.04)	43 (2.36)	564 (8.45)	
**CVD (*n*, %)**				< 0.0001
No	6,436 (91.97)	1,325 (97.27)	5,111 (90.37)	
Yes	748 (8.03)	57 (2.73)	691 (9.63)	
**Take anti-hypertensive or lipid-lowering medication (*n*, %)**				< 0.0001
No	4,557 (67.11)	1,186 (86.47)	3,371 (61.25)	
Yes	2,627 (32.89)	196 (13.53)	2,431 (38.75)	
eGFR (mL/min/1.73 m^2^)	93.49 ± 0.42	97.74 ± 0.72	92.21 ± 0.43	< 0.0001
uACR (mg/g)	30.36 ± 2.92	12.76 ± 1.38	35.69 ± 3.75	< 0.0001
LE8	68.39 ± 0.31	86.76 ± 0.20	62.84 ± 0.23	< 0.0001
Diet score	38.95 ± 0.60	59.15 ± 1.05	32.85 ± 0.57	< 0.0001
Physical activity score	72.11 ± 0.69	94.41 ± 0.58	65.37 ± 0.77	< 0.0001
Nicotine exposure score	71.67 ± 0.74	92.73 ± 0.61	65.31 ± 0.86	< 0.0001
Sleep health score	83.93 ± 0.43	92.83 ± 0.46	81.24 ± 0.50	< 0.0001
Body mass index score	60.41 ± 0.64	84.95 ± 0.76	52.99 ± 0.64	< 0.0001
Blood lipids score	63.78 ± 0.53	82.29 ± 0.80	58.18 ± 0.60	< 0.0001
Blood glucose score	86.25 ± 0.34	97.12 ± 0.42	82.96 ± 0.40	< 0.0001
Blood pressure score	69.96 ± 0.55	90.35 ± 0.60	63.79 ± 0.62	< 0.0001

NHANES 2005–2018. CVH, cardiovascular health; LE8, Life’s Essential 8; PIR, the ratio of family income to poverty; CVD, cardiovascular diseases; eGFR, estimated glomerular filtration rate; uACR, urinary albumin, and creatinine.

### 3.2 PFAS concentration distribution

**Figure 1:**
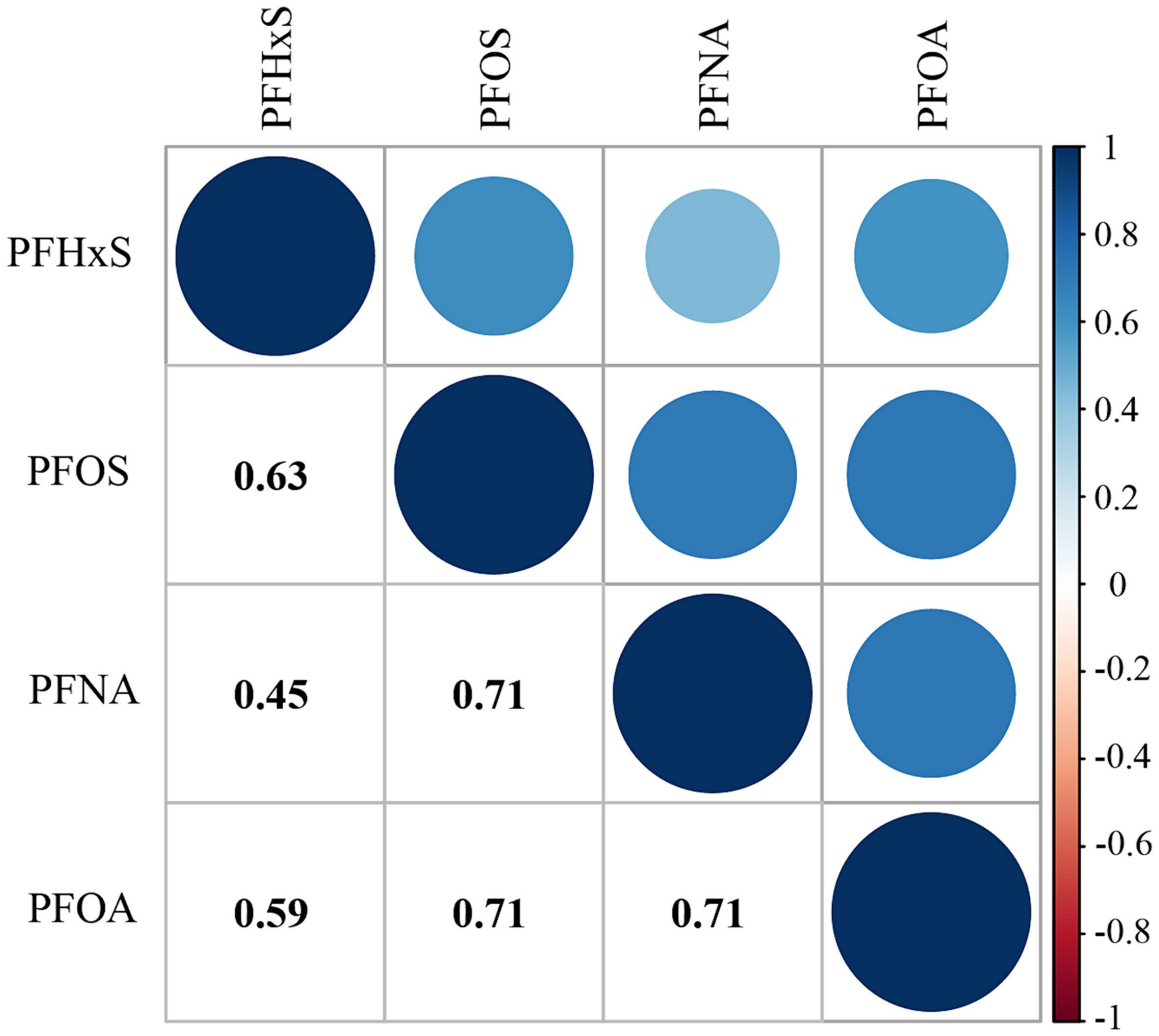
Correlations among the serum PFAS concentrations.

Table 2 shows the distribution of PFAS concentrations. All PFAS compounds investigated had detection rates above 98%, with PFOS being the highest at 100%. PFOS had the highest median serum concentration at 8.40 ng/mL, whereas PFNA had the lowest at 0.90 ng/mL. The median concentrations of PFNA and PFOA were 1.50 ng/mL and 2.57 ng/mL, respectively. The concentrations of PFAS were lower in individuals with high CVH than in those with impaired CVH (*p* < 0.001). Supplementary Table S2 lists the minimum detection concentrations for each PFAS. Additionally, a noticeable downward trend in the median concentrations of each PFAS over the years was observed (*p* < 0.001), as detailed in Supplementary Table S3. Figure 1 shows the results of the Spearman rank correlation analysis, with correlation coefficients ranging from 0.45 to 0.71 for the four PFASs.

**Table 2:** Distribution of PFAS concentrations by cardiovascular health status.

PFAS (ng/mL)	Total	Detection Rate^a^	High CVH	Impaired CVH	*p*-value
PFHxS Median (IQR)	1.50 (0.89, 2.60)	98.51%	1.37 (0.75, 2.35)	1.50 (0.90, 2.70)	< 0.0001
PFNA Median (IQR)	0.90 (0.52, 1.39)	98.30%	0.80 (0.50, 1.23)	0.90 (0.57, 1.40)	< 0.001
PFOA Median (IQR)	2.57 (1.57, 4.20)	100%	2.40 (1.47, 3.70)	2.63 (1.60, 4.30)	< 0.001
PFOS Median (IQR)	8.40 (4.60, 14.90)	99.80%	6.94 (4.00, 12.00)	9.00 (4.80, 15.90)	< 0.0001

^**a**^Detection rates for PFOA and PFOS were calculated for 2005–2012 cycles, because in cycles 2013–2018 PFOA and PFOS were measured as isomers. PFOA, perfluorooctanoic acid; PFOS, perfluorooctane sulfonic acid; PFNA, perfluorononanoic acid; PFHxS, perfluorohexane sulfonic acid.

### 3.3 PFAS exposure and impaired CVH

Table 3 shows weighted logistic regression analyses for variables related to impaired CVH. Age was positively associated with impaired CVH. Men were 56% more likely to have impaired CVH than women were. (OR: 1.56; 95% CI: 1.34, 1.82). There were notable racial differences, with non-Hispanic white, Mexican American, and other individuals showing lower odds of impaired CVH than non-Hispanic black individuals. An inverse relationship between education level and impaired CVH was demonstrated. Lifestyle factors also showed significant associations, with former drinkers and uninsured individuals being at greater risk. Health conditions such as diabetes, depression, and CVD are linked to an increased likelihood of impaired CVH. In particular, each unit increase in the eGFR was shown to have a protective effect against impaired CVH.

**Table 3:** Weighted univariate logistic regression analyses of variables associated with impaired CVH.

Variable	OR	95% CI	*p*-value
Age (years)	1.03	1.03 (1.02, 1.03)	<0.0001
**Gender**			
Female	Reference	Reference	Reference
Male	1.56	1.56 (1.34, 1.82)	<0.0001
**Race/ethnicity**			
Non-Hispanic Black	Reference	Reference	Reference
Non-Hispanic white	0.42	0.42 (0.35, 0.51)	<0.0001
Mexican American	0.51	0.51 (0.40, 0.66)	<0.0001
Other	0.37	0.37 (0.29, 0.48)	<0.0001
**Education levels**			
High school or less	Reference	Reference	Reference
Some college or associate degree	0.51	0.51 (0.41, 0.63)	<0.0001
College graduate or above	0.19	0.19 (0.15, 0.23)	<0.0001
**Marital status**			
Coupled	Reference	Reference	Reference
Single or separated	1.22	1.22 (1.02, 1.44)	0.03
**PIR**			
<1.3	Reference	Reference	Reference
1.3–3.5	0.76	0.76 (0.62, 0.94)	0.01
>3.5	0.44	0.44 (0.36, 0.54)	<0.0001
**Alcohol intake status**			
Never	Reference	Reference	Reference
Former	2.6	2.60 (1.97, 3.44)	<0.0001
Mild	1.1	1.10 (0.88, 1.37)	0.40
Moderate	0.96	0.96 (0.74, 1.26)	0.77
Heavy	1.58	1.58 (1.18, 2.11)	0.002
**Health insurance**			
No	Reference	Reference	Reference
Yes	0.7	0.70 (0.57, 0.85)	<0.001
**Diabetes**			
No	Reference	Reference	Reference
Yes	8.68	8.68 (4.94, 15.26)	<0.0001
**Depression**			
No	Reference	Reference	Reference
Yes	3.83	3.83 (2.51, 5.84)	<0.0001
**CVD**			
No	Reference	Reference	Reference
Yes	3.8	3.80 (2.74, 5.28)	<0.0001
**Take anti-hypertensive or lipid-lowering medication**			
No	Reference	Reference	Reference
Yes	4.04	4.04 (3.29, 4.96)	<0.0001
eGFR (mL/min/1.73 m^2^)	0.99	0.99 (0.98, 0.99)	<0.0001
uACR (mg/g)	1	1.00 (1.00, 1.01)	0.05

CVH, cardiovascular health; PIR, the ratio of family income to poverty; CVD, cardiovascular diseases; eGFR, estimated glomerular filtration rate; uACR, urinary albumin and creatinine.

Table 4 shows the results of weighted logistic regression analyses for each PFAS compound on impaired CVH. In the crude model, the continuous variables PFHxS, PFNA, PFOA, and PFOS were positively associated with impaired CVH (OR_ln-PFHxS_: 1.18, 95% CI: 1.10, 1.27; OR_ln-PFNA_: 1.22, 95% CI: 1.10, 1.35; OR_ln-PFOA_: 1.21, 95% CI: 1.09, 1.33; OR_ln-PFOS_: 1.30, 95% CI: 1.20, 1.41). Categorizations of PFAS with Q1 as a reference revealed that higher quartiles, especially Q4, were associated with an increased risk of impaired CVH, trend test *p* < 0.001 (OR_PFHxS_: 1.63, 95% CI: 1.33, 1.99; OR_PFNA_: 1.54, 95% CI: 1.21, 1.96; OR_PFOA_: 1.56, 95% CI: 1.24, 1.95; OR_PFOS_: 2.20, 95% CI: 1.72, 2.81).

**Table 4:** Results of weighted multivariable logistic regression analyses for PFAS compounds and impaired CVH.

PFAS exposure (ng/ml)	Crude model			Model 1		
	OR	95% CI	*P*-value	OR	95% CI	*P*-value
**PFHxS**						
ln-PFHxS	1.18	1.10, 1.27	<0.0001	1.07	0.99, 1.17	0.09
Q1 (low)	1	Reference		1	Reference	
Q2	1.48	1.20, 1.83	<0.001	1.33	1.05, 1.69	0.02
Q3	1.38	1.10, 1.73	0.01	1.09	0.85, 1.40	0.49
Q4 (high)	1.63	1.33, 1.99	<0.0001	1.21	0.95, 1.54	0.13
p for trend			<0.0001			0.33
**PFNA**						
ln-PFNA	1.22	1.10, 1.35	<0.001	1.14	1.02, 1.28	0.03
Q1 (low)	1	Reference		1	Reference	
Q2	1.08	0.89, 1.32	0.41	1.13	0.91, 1.39	0.28
Q3	1.30	1.04, 1.62	0.02	1.22	0.97, 1.54	0.09
Q4 (high)	1.54	1.21, 1.96	<0.001	1.40	1.09, 1.80	0.01
p for trend			<0.001			0.01
**PFOA**						
ln-PFOA	1.21	1.09, 1.33	<0.001	1.15	1.02, 1.29	0.02
Q1 (low)	1	Reference		1	Reference	
Q2	1.14	0.93, 1.40	0.2	1.13	0.91, 1.41	0.25
Q3	1.18	0.94, 1.48	0.15	1.14	0.88, 1.49	0.32
Q4 (high)	1.56	1.24, 1.95	<0.001	1.44	1.10, 1.88	0.01
p for trend			<0.001			0.02
**PFOS**						
ln-PFOS	1.30	1.20, 1.41	<0.0001	1.18	1.08, 1.30	<0.001
Q1 (low)	1	Reference		1	reference	
Q2	1.07	0.85, 1.34	0.58	0.98	0.78, 1.24	0.86
Q3	1.44	1.14, 1.81	0.002	1.27	1.01, 1.59	0.04
Q4 (high)	2.20	1.72, 2.81	<0.0001	1.62	1.25, 2.11	<0.001
p for trend			<0.0001			<0.0001

Crude model: no covariates were adjusted. Model 1: age (as a continuous variable), gender, race/ethnicity, educational level, marital status, PIR, alcohol consumption, health insurance, diabetes, depression, take anti-hypertensive or lipid-lowering medication, cardiovascular disease, eGFR, and UACR. PFOA, perfluorooctanoic acid; PFOS, perfluorooctane sulfonic acid; PFNA, perfluorononanoic acid; PFHxS, perfluorohexane sulfonic acid. OR, odds ratio; 95% CI, confidence intervals.

### 3.4 Dose–response relationship between PFAS and impaired CVH

**Figure 2:**
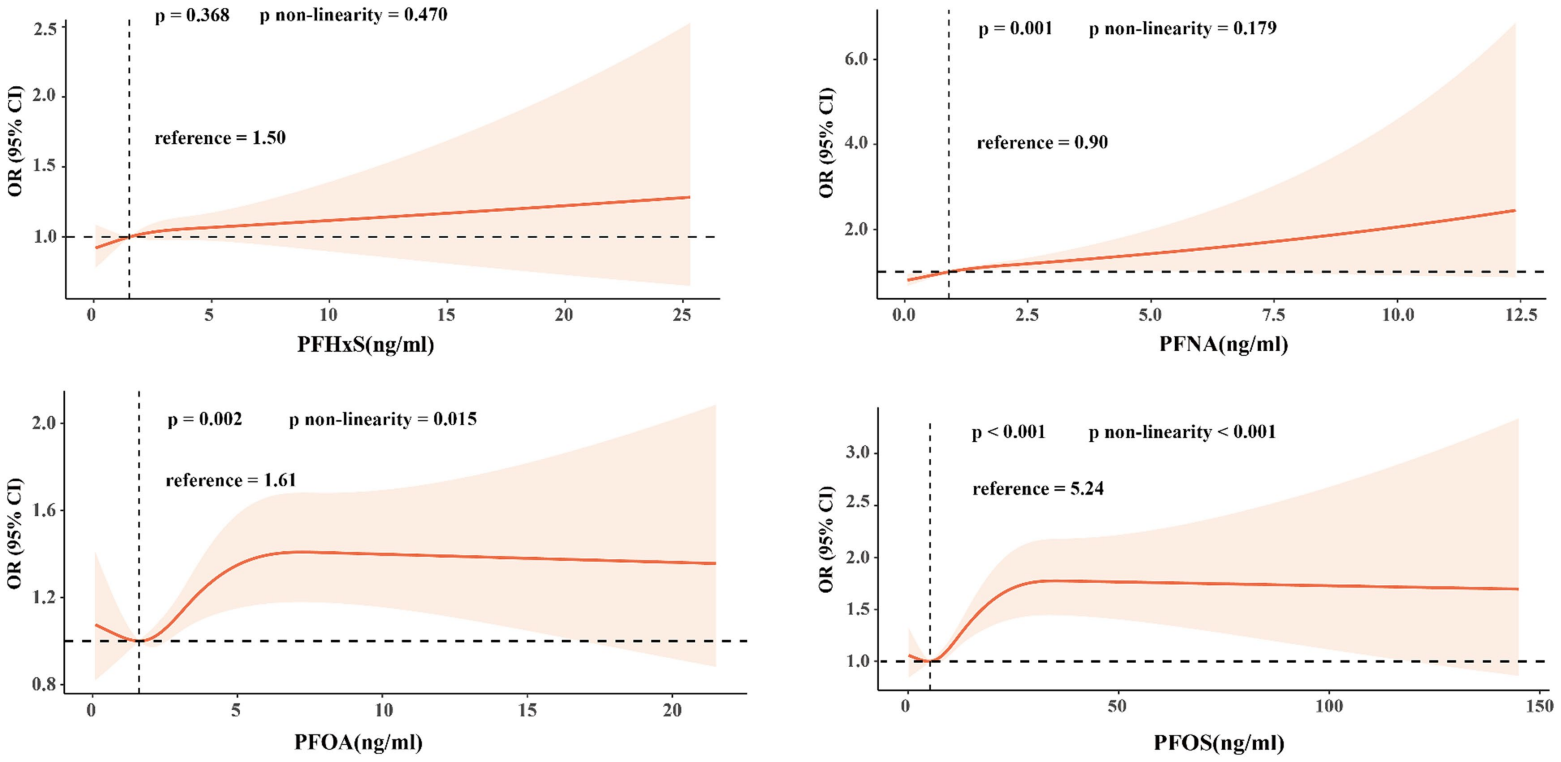
Restricted cubic splines were used to determine the relationship between PFAS exposure concentration and the risk of impaired CVH in adults.

Figure 2 shows the RCS curves for each PFAS in relation to impaired CVH, adjusting for the covariates described in Model 1. There was a linear relationship between PFNA levels and impaired CVH (*p* = 0.001; p for nonlinearity = 0.179). In contrast, the serum PFOA and PFOS levels exhibited a nonlinear relationship with impaired CVH (p for nonlinearity = 0.015 and p for nonlinearity <0.001, respectively). The minimum thresholds for a favorable association were identified at 1.61 ng/mL for PFOA and 5.24 ng/mL for PFOS.

### 3.5 Subgroup analysis

Supplementary Figures S2–S5 show the subgroup analysis results. A positive association was noted between each PFAS exposure and impaired CVH in the 20–39 age cohort. In the 40–59 age group, PFHxS, PFOA, and PFOS were all positively associated with impaired CVH, whereas in the 60+ age group, PFOS exposure was of particular concern. Among males, PFNA and PFOS exposure were positively associated with impaired CVH, with similar caution advised for females regarding PFOS. Non-Hispanic white individuals were positively associated with impaired CVH for all four PFAS compounds, and among Mexican Americans, PFHxS, PFNA, and PFOS were all positively associated with CVH impairment. In terms of education, PFOS exposure was consistently associated with impaired CVH across all levels of education, with notable concern also directed at PFHxS exposure among those with less than a high school education and a college degree or above and PFOA and PFNA exposure among those with a college degree or above. With respect to economic status, as measured by PIR, serum PFAS levels were predominantly associated with impaired CVH at 1.3–3.5 and 3.5 above the PIR range. For marital status, all four PFAS compounds were associated with a greater risk of impaired CVH in coupled individuals, a trend also observed among those with health insurance. Interaction analyses revealed that race/ethnicity influenced the associations between PFHxS and PFOS exposure and impaired CVH. Marital status influenced the associations between PFOA and PFOS exposure and impaired CVH.

### 3.6 WQS and Qgcomp analysis

**Figure 3:**
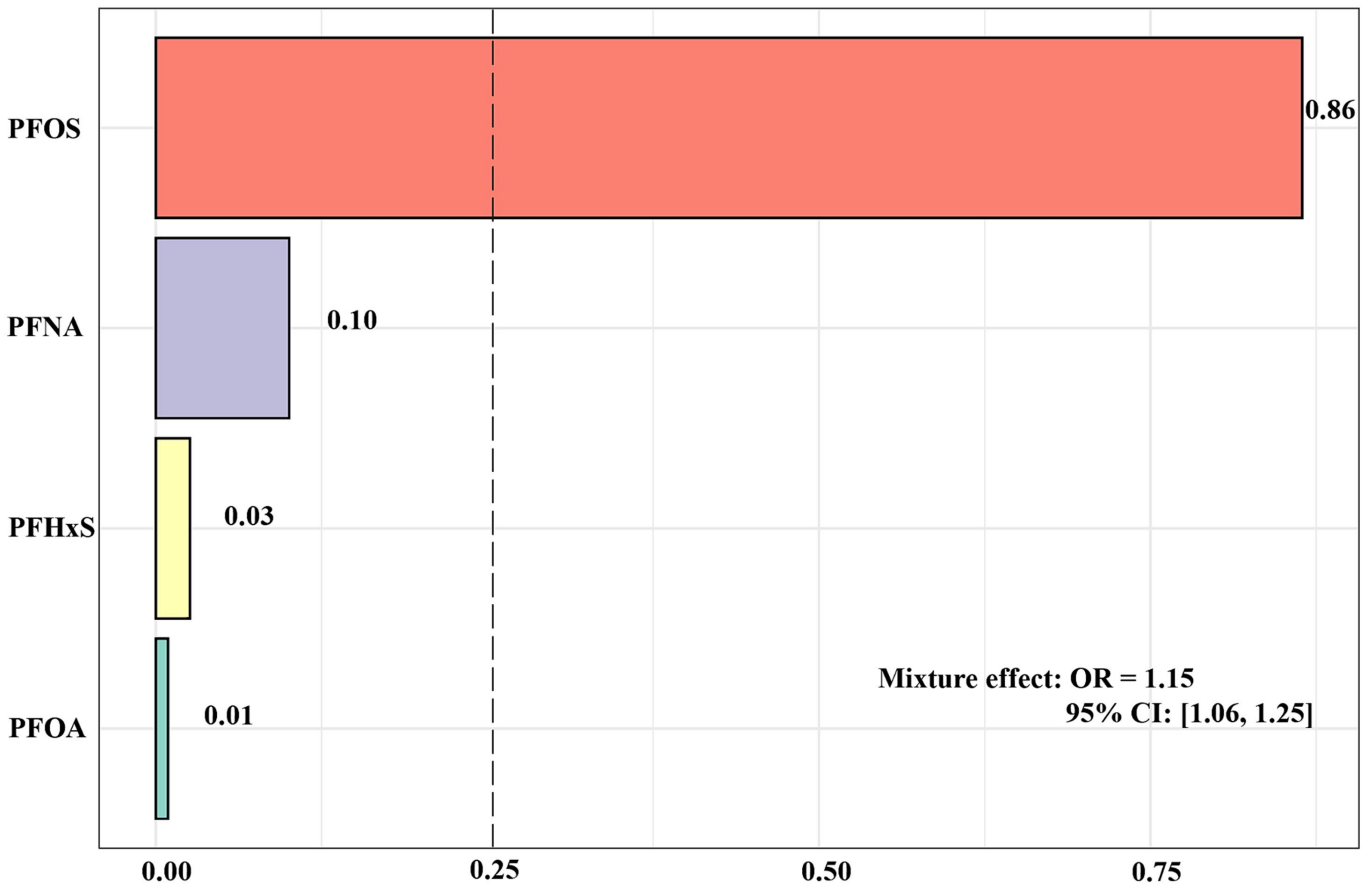
WQS regression index weights for each PFAS in the PFAS mixture.

Figure 3 shows the results of the analysis of the WQS model. The results of the WQS via a positive model revealed that each quartile increase in mixed PFASs was associated with an increased risk of impaired CVH (OR: 1.15, 95% CI: 1.06, 1.25), with PFOS having the largest positive weight (0.86). In contrast to WQS, Qgcomp allows weights to move in either direction, reflecting the complex interplay within the mixture. The analysis conducted by Qgcomp revealed that each quantile increase in the serum concentration of all PFASs was associated with increased odds of impaired CVH (OR: 1.10, 95% CI: 1.02, 1.19). In particular, PFOS and PFNA contributed the main positive weights to the outcome, with PFOS having the largest positive weight (0.61) and PFNA following 0.39; PFHxS and PFOA had negative weights, with PFHxS having the largest negative weight at 0.93 and PFOA having a negative weight of 0.07 (Supplementary Figure S6).

### 3.7 Sensitivity analysis

Supplementary Table S4 shows the outcomes of the sensitivity analysis. The exposure levels of PFNA, PFOA, and PFOS in the highest quartile were still positively associated with impaired CVH (OR_PFNA_: 1.49, 95% CI: 1.14, 1.94; OR_PFOA_: 1.50, 95% CI: 1.13, 1.98; OR_PFOS_: 1.74, 95% CI: 1.30, 2.33). Similarly, ln-PFNA, ln-PFOA, and ln-PFOS were found to be associated with an increased risk of impaired CVH (OR_ln-PFNA_: 1.18, 95% CI: 1.04, 1.33; OR_ln-PFOA_: 1.17, 95% CI: 1.03, 1.32; OR_ln-PFOS_: 1.21, 95% CI: 1.09, 1.34).

## 4 Discussions

In this nationally representative study, we determined that PFAS were associated with a greater risk of impaired CVH. After controlling for covariates, the risk of impaired CVH was increased by 40, 10, and 25% for PFNA, PFOA, and PFOS, respectively, in the highest group compared with the lowest group. Similar statistical results were obtained when PFAS was used as a continuous variable. Trend tests validated the persistence of the positive relationships, and subgroup analysis results underscored the universality of this association across different subpopulations. The sensitivity analysis reinforced the robustness of the statistical results. The RCS curve revealed that PFNA maintains a positive linear relationship with impaired CVH, whereas PFOS and PFOA exhibit a nonlinear relationship with impaired CVH. The results of the Qgcomp analysis and WQS model revealed that mixed exposure to PFASs is linked to impaired CVH. PFOS had the most positive weight, as did PFHxS, which had the largest negative weight.

Previous studies have linked serum PFAS exposure and CVD, such as coronary heart disease, stroke, carotid artery thickness, and lower limb arterial occlusion (11, 26). These investigations relied primarily on medical history for diagnosis and lacked a direct focus on CVH. Given that young people have a lower risk of CVD, we used LE8 to examine the associations between PFASs and CVH and included a wider range of health indicators.

In the present study, exposure to almost all PFOS was positively associated with impaired CVH in the young and middle-aged groups. The association was particularly significant in the middle-aged group. This possibly reflects the cumulative effect of chronic exposure with age, as confirmed by Supplementary Table S5, which shows the cumulative effect of increasing PFAS concentrations with age. In contrast, for older adults aged 60 years and older, despite having longer cumulative exposure concentrations, only the presence of an effect of PFOS exposure on impaired CVH was found, hinting at a potential relevance of age, which is in line with the findings of a previous German study that reported that, relative to older adults, PFAS have a greater risk of increasing cardiometabolic outcomes in people younger than 54 years (16). Although the physiological mechanisms are not understood, multiple CVH risk factors are already present in older adults, including metabolic abnormalities, medications, and ageing; these issues increase the degree of competing risk and may mask the independent effects of PFASs. Our finding that exposure to PFNA and PFOS was more significantly associated with impaired CVH in males than in females suggests that there may be biological or environmental factors that increase the susceptibility of males to the negative effects of these chemicals on CVH. Consistent with our results, Sun and colleagues reported higher serum PFAS concentrations in men than in women (25). Pan and colleagues reported that males exposed to PFNA, PFOA, and PFOS have a greater risk of hypertension than females do, which may be influenced by differences in hormone levels and body clearance by sex (27). Non-Hispanic whites and Mexican Americans are more susceptible to PFAS for CVH. According to Liddie et al. (28), the detection of PFASs in community water systems in the U.S. was positively associated with the number of sources of PFASs and the proportion of people of color served by these water systems, highlighting the role of race in environmental health inequities. Additionally, our study revealed a linear relationship between PFNA levels and impaired CVH, suggesting that the risk increases with increasing exposure. In contrast, there was a nonlinear trend for PFOA and PFOS, with the ratio of impaired CVH increasing as concentrations reached a certain threshold, beyond which the risk stabilized. These findings suggest that complex biological mechanisms may reduce risk at higher exposures. Individuals who are coupled have an increased risk of impaired CVH risk following exposure to four PFASs, and further research may be needed to investigate the relationships between different lifestyles and environmental factors and PFASs and CVH. Given the cross-sectional study design, it is important to consider that high CVH might enhance PFAS elimination. The HOME study involving 166 mother–child pairs revealed that physical exercise modified the impact of PFOA exposure on cardiac metabolic risk scores, visceral fat area, and insulin resistance, suggesting that lifestyle interventions could mitigate some adverse effects of PFAS exposure (29).

The mechanism by which PFASs affect CVH is not fully understood. Previous evidence has suggested that PFAS may be associated with the aggravation of CVD risk factors and events through endocrine disruption and possibly a direct vascular toxic effect (30). Endothelial dysfunction is widely recognized as a foundational pathology of various CVDs. Earlier experimental research suggested that PFAS exposure can induce inflammation, initiate the generation of reactive oxygen species, increase endothelial cell permeability, and increase the expression of adhesion molecules, such as intercellular adhesion molecule-1, thus attracting monocytes to atherosclerotic lesions and exacerbating atherosclerosis (31, 32). Omoike et al. (33) reported that PFAS exposure was linked to increased serum markers of chronic inflammation and oxidative stress, such as lymphocyte count, serum iron, albumin, and bilirubin. In animal experiments, PFAS has been found to cause cardiotoxicity in rats by increasing cell apoptosis and proinflammatory cytokine expression (34). DNA methylation, an important epigenetic modification predominantly occurring on cytosine–phosphate–guanine islands in gene promoter regions and regulated by DNA methyltransferases, has recently been connected to the expression of genes linked to CVD (35). Research indicates that PFAS exposure is correlated with epigenetic alterations, including DNA methylation, in adults and birth cohorts (36, 37). Lin’s study revealed an association between PFOS exposure and elevated 5mdC/dG levels, highlighting the potential relevance of DNA methylation in the pathophysiology of PFOS-related atherosclerosis (38). Another potential pathway involves PPAR receptor activation, which is crucial for fatty acid and metabolism regulation and is a potential drug target for reducing atherosclerosis risk; however, this pathway is associated with increased cardiovascular events (39, 40). PFAS interaction with PPARs has been shown to lead to hypertension (41). Additionally, PFOS preferentially accumulates in platelets, affecting the stability of the plasma membrane and altering membrane fluidity, which affects platelet activation and aggregation, leading to thrombosis (42).

This study is the first to measure and diagnose impaired CVH risk associated with PFASs via the LE8 score. This method has several advantages. First, we use NHANES data to ensure data quality and national representativeness by adjusting for appropriate weights and confounders. Second, the LE8 score is employed as a comprehensive CVH assessment criterion. Directly linking PFAS exposure to quantifiable changes in CVH facilitates early identification of at-risk individuals. Third, applying multiple statistical analysis techniques strengthens the robustness of the findings by identifying specific subpopulations and elucidating complex relationships.

Our study has several clear limitations. First, we only analyzed data from a single measurement of serum PFAS concentrations rather than from repeated measurements, which would be more appropriate for estimating the cumulative effects of PFAS exposure over many years. Second, since certain metrics are derived from self-report questionnaires, this may introduce potential biases. Third, although our study controlled for a wide range of confounding factors, there are still potential confounding factors that could affect the results. Finally, we cannot determine the inherent causal relationships given the limitations of the study type.

## 5 Conclusion

In summary, our research revealed a significant association between PFAS exposure and elevated risk of impaired CVH, with almost consistent results across diverse subpopulations. These findings contribute to the advancement of understanding PFAS risks in public health and environmental studies, to identify individuals at risk before major health events occur and potentially alleviate the overall burden of diseases in the population. Furthermore, there is a pressing need for future longitudinal studies on populations with high PFAS exposure and emerging PFAS compounds to confirm the current findings. Finally, in-depth experimental research is essential to uncover the potential mechanisms behind this association.

## Data Availability

Publicly available datasets were analyzed in this study. This data can be found at: https://wwwn.cdc.gov/nchs/nhanes/continuousnhanes/default.aspx.
