# Supplementary material for: Exposure to per- and polyfluoroalkyl substances is associated with impaired cardiovascular health: a cross-sectional study
Source: Front Public Health. 2024 Aug 29;12:1418134. doi: 10.3389/fpubh.2024.1418134 (PMC11390656; doi:10.3389/fpubh.2024.1418134)
Supplement: Supplementary file 1 [file Data_Sheet_1.docx]

Supplementary Material

# Supplementary Figures and Tables

## List of Figures and Tables:

**Supplementary Table 1:** Definition and scoring approach for the American Heart Association’s Life’s Essential 8 score.

**Supplementary Table 2:** Limits of detection (ng/mL) for serum per- and polyfluoroalkyl substance concentrations across cycles, NHANES 2005-2018.

**Supplementary Table 3:** The time trend of serum PFAS concentration (ng/ml) in NHANES subjects from 2005 to 2018.

**Supplementary Table 4:** Weighted logistic regression analysis of the association between PFAS exposure and impaired CVH after excluding participants with cardiovascular diseases, diabetes, and depression.

**Supplementary Table 5:** Distribution of PFAS concentrations in different subgroups.

**Supplementary Table 6:** STROBE Statement—Checklist of items that should be included in reports of ***cross-sectional studies***.

**Supplementary Figure 1:** Flow chart illustrating the inclusion and exclusion criteria for the study participants.

**Supplementary Figure 2:** Subgroup and interaction analyses of PFHxS and impaired CVH.

**Supplementary Figure 3:** Subgroup and interaction analyses of PFNA and impaired CVH.

**Supplementary Figure 4:** Subgroup and interaction analyses of PFOA and impaired CVH.

**Supplementary Figure 5:** Subgroup and interaction analyses of PFOS and impaired CVH.

**Supplementary Figure 6:** Quantile g-computation scaled weights for each PFAS in the PFAS mixture.

## Supplementary Tables:

**Supplementary Table 1.** Definition and scoring approach for the American Heart Association’s Life’s Essential 8 score.

| **Domain** | **CVH Metric** | **Method of measurement** | **Quantification and Scoring of CVH Metric** |
| --- | --- | --- | --- |
| **Health Behaviors** | Diet | Healthy Eating Index-2015 diet score percentile | Quantiles of HEI-2015 diet (Population)  Scoring (Population):  Points Quantile  100 ≥95^th^ percentile (top/ideal diet)  80 75^th^ – 94^th^ percentile  50 50^th^ – 74^th^ percentile  25 25^th^ – 49^th^ percentile  0 1^st^ – 24^th^ percentile (bottom/least ideal quartile) |
|  | PA | Self-reported minutes of moderate or vigorous physical activity per week | Metric: Minutes of moderate (or greater) intensity activity per week  Scoring:  Point Minutes  100 ≥150  90 120 – 149  80 90 – 119  60 60 – 89  40 30 – 59  20 1 – 29  0 0 |
|  | Nicotine exposure | Self-reported use of cigarettes or inhaled nicotine-delivery system (NDS) | Metric: Combustible tobacco use or inhaled NDS use; or secondhand smoke exposure  Scoring:  Points Status  100 Never smoker  75 Former smoker, quit ≥5 years  50 Former smoker, quit 1 - <5 years  25 Former smoker, quit <1 year, or currently using inhaled NDS  0 Current smoker  Subtract 20 points (unless score is 0) for living with active indoor smoker in home |
|  | Sleep health | Self-reported average hours of sleep per night | Metric: Average hours of sleep per night  Scoring:  Points Level  100 7 – <9  90 9 – <10  70 6 – <7  40 5 – <6 or ≥ 10  20 4 – <5  0 <4 |
| **Health Factors** | Body mass index | Body weight (kg) divided by height squared (m^2^) | Metric: Body mass index (kg/m^2^)  Scoring**:**  Points Level  100 <25  70 25.0 – 29.9  30 30.0 – 34.9  15 35.0 – 39.9  0 ≥40.0 |
|  | Blood lipids | Plasma total and HDL-cholesterol with calculation of non-HDL-cholesterol | Metric: Non-HDL-cholesterol (mg/dL)  Scoring:  Points Level  100 <130  60 130 – 159  40 160 – 189  20 190 – 219  0 ≥220  If drug-treated level, subtract 20 points |
|  | Blood glucose | Fasting blood glucose or casual HbA1c | Metric: Fasting blood glucose (mg/dL) or HbA1c (%)  Scoring:  Points Level  100 No history of diabetes and FBG <100 (or HbA1c < 5.7)  60 No diabetes and FBG 100 – 125 (or HbA1c 5.7-6.4) (prediabetes)  40 Diabetes with HbA1c <7.0  30 Diabetes with HbA1c 7.0 – 7.9  20 Diabetes with HbA1c 8.0 – 8.9  10 Diabetes with Hb A1c 9.0 – 9.9  0 Diabetes with HbA1c ≥10.0 |
|  | Blood pressure | Appropriately measured systolic and diastolic blood pressure | Metric: Systolic and diastolic blood pressure (mm Hg)  Scoring:  Points Level  100 <120/<80 (optimal)  75 120-129/<80 (elevated)  50 130-139 or 80-89 (Stage I HTN)  25 140-159 or 90-99  0 ≥160 or ≥100  Subtract 20 points if treated level |

**Supplementary Table 2.** Limits of detection (ng/mL) for serum per- and polyfluoroalkyl substance concentrations across cycles, NHANES 2005-2018.

|  | **PFNA** | **PFHxS** | **PFOS** | **PFOA** |
| --- | --- | --- | --- | --- |
| 2005-2006 | 0.1 | 0.1 | 0.2 | 0.1 |
| 2007-2008 | 0.082 | 0.1 | 0.2 | 0.1 |
| 2009-2010 | 0.082 | 0.1 | 0.2 | 0.1 |
| 2011-2012 | 0.08 | 0.1 | 0.2 | 0.1 |
| 2013-2014^a^ | 0.1 | 0.1 | 0.2 | 0.1 |
| 2015-2016^a^ | 0.1 | 0.1 | 0.1 | 0.1 |
| 2017-2018^a^ | 0.1 | 0.1 | 0.1 | 0.1 |

Abbrev: PFOA, perfluorooctanoic acid; PFOS, perfluorooctane sulfonic acid; PFNA, perfluorononanoic acid; PFHxS, perfluorohexane sulfonic acid.

a: The limits of detection for PFOA and PFOS isomers in cycles of 2013-2018 were all 0.1 ng/mL.

**Supplementary Table 3.** The time trend of serum PFAS concentration (ng/ml) in NHANES subjects from 2005 to 2018.

| **PFAS (ng/mL)** | **PFHxS** | **PFNA** | **PFOA** | **PFOS** |
| --- | --- | --- | --- | --- |
| Total | 1.50(0.89,2.60) | 0.90(0.52,1.39) | 2.57(1.57,4.20) | 8.40(4.60,14.90) |
| 2005-2006 | 1.70(1.00,3.10) | 1.10(0.70,1.70) | 4.20(2.80,6.50) | 17.90(11.70,27.90) |
| 2007-2008 | 2.00(1.10,3.50) | 1.23(0.90,1.72) | 4.40(3.10,6.20) | 14.30(9.10,21.80) |
| 2009-2010 | 1.70(1.00,2.90) | 1.23(0.90,1.80) | 3.30(2.30,4.70) | 10.50(6.50,15.70) |
| 2011-2012 | 1.31(0.77,2.30) | 0.91(0.62,1.35) | 2.23(1.51,3.23) | 7.40(4.41,11.30) |
| 2013-2014 | 1.40(0.80,2.60) | 0.70(0.50,1.00) | 2.17(1.37,3.17) | 5.70(3.30, 9.10) |
| 2015-2016 | 1.20(0.80,2.10) | 0.60(0.40,0.90) | 1.67(1.07,2.47) | 5.30(3.10, 8.60) |
| 2017-2018 | 1.20(0.70,2.00) | 0.50(0.30,0.80) | 1.57(1.07,2.37) | 4.90(2.70, 8.20) |
| P-value | < 0.0001 | < 0.0001 | < 0.0001 | < 0.0001 |

Abbrev: PFOA, perfluorooctanoic acid; PFOS, perfluorooctane sulfonic acid; PFNA, perfluorononanoic acid; PFHxS, perfluorohexane sulfonic acid.

**Supplementary Table 4.** Weighted logistic regression analysis of the association between PFAS exposure and impaired CVH after excluding participants with cardiovascular diseases, diabetes, and depression.

| **PFAS exposure (ng/ml)** | **Crude model** | | **Model 1** | |
| --- | --- | --- | --- | --- |
|  | **OR (95% CI)** | **P-Value** | **OR (95% CI)** | **P-Value** |
| **PFHXS** |  |  |  |  |
| ln-PFHxS | 1.22(1.12,1.32) | <0.0001 | 1.08(0.99,1.18) | 0.09 |
| Q1 (low) | ref |  | ref |  |
| Q2 | 1.50(1.19,1.88) | <0.001 | 1.29(0.99,1.67) | 0.06 |
| Q3 | 1.51(1.20,1.91) | <0.001 | 1.14(0.89,1.46) | 0.31 |
| Q4 (high) | 1.70(1.37,2.12) | <0.0001 | 1.20(0.94,1.55) | 0.15 |
| p for trend |  | <0.0001 |  | 0.26 |
| **PFNA** |  |  |  |  |
| ln-PFNA | 1.27(1.15,1.40) | <0.0001 | 1.18(1.04,1.33) | 0.01 |
| Q1 (low) | ref |  | ref |  |
| Q2 | 1.17(0.96,1.43) | 0.12 | 1.19(0.94,1.49) | 0.14 |
| Q3 | 1.39(1.10,1.76) | 0.01 | 1.31(1.03,1.68) | 0.03 |
| Q4 (high) | 1.71(1.34,2.18) | <0.0001 | 1.49(1.14,1.94) | 0.003 |
| p for trend |  | <0.0001 |  | 0.002 |
| **PFOA** |  |  |  |  |
| ln-PFOA | 1.28(1.16,1.42) | <0.0001 | 1.17(1.03,1.32) | 0.01 |
| Q1 (low) | ref |  | ref |  |
| Q2 | 1.16(0.93,1.44) | 0.18 | 1.10(0.86,1.41) | 0.44 |
| Q3 | 1.23(0.98,1.54) | 0.08 | 1.16(0.88,1.52) | 0.29 |
| Q4 (high) | 1.74(1.39,2.19) | <0.0001 | 1.50(1.13,1.98) | 0.01 |
| p for trend |  | <0.0001 |  | 0.01 |
| **PFOS** |  |  |  |  |
| ln-PFOS | 1.35(1.24,1.48) | <0.0001 | 1.21(1.09,1.34) | <0.001 |
| Q1 (low) | ref |  | ref |  |
| Q2 | 1.09(0.87,1.38) | 0.45 | 0.98(0.77,1.24) | 0.86 |
| Q3 | 1.44(1.14,1.82) | 0.003 | 1.27(0.99,1.61) | 0.06 |
| Q4 (high) | 2.41(1.86,3.11) | <0.0001 | 1.74(1.30,2.33) | <0.001 |
| p for trend |  | <0.0001 |  | <0.0001 |

Adjust for age (as a continuous variable), gender, race/ethnicity, educational level, marital status, PIR, alcohol consumption, health insurance, take anti-hypertensive or lipid-lowering medication, eGFR, and UACR.

Abbreviations: PFOA, perfluorooctanoic acid; PFOS, perfluorooctane sulfonic acid; PFNA, perfluorononanoic acid; PFHxS, perfluorohexane sulfonic acid. OR, odds ratio; 95% CI,confidence intervals.

**Supplementary Table 5.** Distribution of PFAS concentrations in different subgroups.

| **PFAS exposure (ng/ml)** | **PFHxS** | **PFNA** | **PFOA** | **PFOS** |
| --- | --- | --- | --- | --- |
| **Age group** |  |  |  |  |
| 20-39 | 1.27(0.70,2.40) | 0.80(0.46,1.21) | 2.27(1.37,3.80) | 6.70(3.50,11.90) |
| 40-59 | 1.40(0.80,2.50) | 0.90(0.57,1.39) | 2.60(1.57,4.20) | 8.30(4.70,14.60) |
| >=60 | 1.90(1.18,3.10) | 0.98(0.66,1.50) | 2.97(1.93,4.60) | 11.10(6.40,19.00) |
| P | < 0.0001 | < 0.0001 | < 0.0001 | < 0.0001 |
| **Gender** |  |  |  |  |
| Female | 1.10(0.60,1.90) | 0.80(0.50,1.30) | 2.27(1.30,3.70) | 6.60(3.43,12.10) |
| Male | 2.00(1.25,3.20) | 0.92(0.60,1.44) | 2.90(1.87,4.70) | 10.40(6.10,17.60) |
| P | < 0.0001 | < 0.0001 | < 0.0001 | < 0.0001 |
| **Race/ethnicity** |  |  |  |  |
| Non-Hispanic Black | 1.30(0.70,2.50) | 0.91(0.57,1.56) | 2.27(1.27,3.72) | 9.40(4.30,18.70) |
| Non-Hispanic white | 1.60(0.96,2.80) | 0.90(0.57,1.39) | 2.77(1.77,4.50) | 9.08(5.00,15.50) |
| Mexican American | 1.20(0.60,1.90) | 0.72(0.40,1.15) | 1.90(1.17,3.17) | 6.00(3.10,10.70) |
| other | 1.20(0.60,2.00) | 0.80(0.50,1.30) | 2.03(1.30,3.20) | 6.40(3.50,11.20) |
| P | < 0.0001 | < 0.0001 | < 0.0001 | < 0.0001 |
| **Education levels** |  |  |  |  |
| High school or less | 1.50(0.90,2.60) | 0.90(0.50,1.39) | 2.47(1.47,4.34) | 8.60(4.50,15.90) |
| Some college or associates degree | 1.50(0.80,2.60) | 0.85(0.50,1.39) | 2.57(1.54,4.10) | 8.20(4.50,14.60) |
| College graduate or above | 1.50(0.90,2.60) | 0.90(0.60,1.39) | 2.67(1.72,4.17) | 8.40(4.70,14.10) |
| P | 0.54 | 0.25 | 0.49 | 0.65 |
| **PIR** |  |  |  |  |
| <1.3 | 1.30(0.70,2.30) | 0.79(0.49,1.20) | 2.17(1.30,3.57) | 7.10(3.60,12.70) |
| 1.3-3.5 | 1.40(0.80,2.50) | 0.82(0.50,1.39) | 2.48(1.47,4.20) | 8.30(4.38,15.10) |
| >3.5 | 1.60(0.90,2.78) | 0.92(0.60,1.40) | 2.77(1.80,4.40) | 9.10(5.10,15.60) |
| P | < 0.0001 | < 0.0001 | < 0.0001 | < 0.0001 |
| **Marital status** |  |  |  |  |
| Coupled | 1.50(0.80,2.60) | 0.90(0.57,1.40) | 2.57(1.57,4.20) | 8.60(4.70,15.30) |
| Single or separated | 1.40(0.90,2.60) | 0.85(0.50,1.30) | 2.60(1.57,4.20) | 8.03(4.40,14.10) |
| P | 0.73 | 0.02 | 0.64 | 0.01 |
| **Health insurance** |  |  |  |  |
| No | 1.40(0.80,2.60) | 0.80(0.50,1.31) | 2.30(1.37,4.00) | 7.70(4.10,13.20) |
| Yes | 1.50(0.90,2.60) | 0.90(0.56,1.39) | 2.60(1.57,4.20) | 8.51(4.70,15.20) |
| P | 0.03 | 0.04 | 0.03 | 0.01 |

Abbreviations: PFOA, perfluorooctanoic acid; PFOS, perfluorooctane sulfonic acid; PFNA, perfluorononanoic acid; PFHxS, perfluorohexane sulfonic acid.

**Supplementary Table 6:** STROBE Statement—Checklist of items that should be included in reports of ***cross-sectional studies***.

|  | Item No | Recommendation | Page No |
| --- | --- | --- | --- |
| **Title and abstract** | 1 | (*a*) Indicate the study’s design with a commonly used term in the title or the abstract | 1 |
|  |  | (*b*) Provide in the abstract an informative and balanced summary of what was done and what was found | 1-2 |
| Introduction | | | |
| Background/rationale | 2 | Explain the scientific background and rationale for the investigation being reported | 2-3 |
| Objectives | 3 | State specific objectives, including any prespecified hypotheses | 3 |
| Methods | | | |
| Study design | 4 | Present key elements of study design early in the paper | 3-4 |
| Setting | 5 | Describe the setting, locations, and relevant dates, including periods of recruitment, exposure, follow-up, and data collection | 3 |
| Participants | 6 | (*a*) Give the eligibility criteria, and the sources and methods of selection of participants | 3 |
| Variables | 7 | Clearly define all outcomes, exposures, predictors, potential confounders, and effect modifiers. Give diagnostic criteria, if applicable | 4-5 |
| Data sources/ measurement | 8* | For each variable of interest, give sources of data and details of methods of assessment (measurement). Describe comparability of assessment methods if there is more than one group | 4-5 |
| Bias | 9 | Describe any efforts to address potential sources of bias | 5-6 |
| Study size | 10 | Explain how the study size was arrived at | 3 |
| Quantitative variables | 11 | Explain how quantitative variables were handled in the analyses. If applicable, describe which groupings were chosen and why | 4-5 |
| Statistical methods | 12 | (*a*) Describe all statistical methods, including those used to control for confounding | 5-6 |
|  |  | (*b*) Describe any methods used to examine subgroups and interactions | 6 |
|  |  | (*c*) Explain how missing data were addressed | 3-4 |
|  |  | (*d*) If applicable, describe analytical methods taking account of sampling strategy | 5-6 |
|  |  | (*e*) Describe any sensitivity analyses | 6 |
| Results | | | |
| Participants | 13* | (a) Report numbers of individuals at each stage of study—eg numbers potentially eligible, examined for eligibility, confirmed eligible, included in the study, completing follow-up, and analysed | 6 |
|  |  | (b) Give reasons for non-participation at each stage | 4 |
|  |  | (c) Consider use of a flow diagram | 4 |
| Descriptive data | 14* | (a) Give characteristics of study participants (eg demographic, clinical, social) and information on exposures and potential confounders | 6-7 |
|  |  | (b) Indicate number of participants with missing data for each variable of interest | 3-4 |
| Outcome data | 15* | Report numbers of outcome events or summary measures | 6-7 |
| Main results | 16 | (*a*) Give unadjusted estimates and, if applicable, confounder-adjusted estimates and their precision (eg, 95% confidence interval). Make clear which confounders were adjusted for and why they were included | 7 |
|  |  | (*b*) Report category boundaries when continuous variables were categorized | 8 |
|  |  | (*c*) If relevant, consider translating estimates of relative risk into absolute risk for a meaningful time period |  |
| Other analyses | 17 | Report other analyses done—eg analyses of subgroups and interactions, and sensitivity analyses | 8-9 |
| Discussion | | | |
| Key results | 18 | Summarise key results with reference to study objectives | 9 |
| Limitations | 19 | Discuss limitations of the study, taking into account sources of potential bias or imprecision. Discuss both direction and magnitude of any potential bias | 11 |
| Interpretation | 20 | Give a cautious overall interpretation of results considering objectives, limitations, multiplicity of analyses, results from similar studies, and other relevant evidence | 9-11 |
| Generalisability | 21 | Discuss the generalisability (external validity) of the study results | 11 |
| Other information | | | |
| Funding | 22 | Give the source of funding and the role of the funders for the present study and, if applicable, for the original study on which the present article is based | 12 |

*Give information separately for exposed and unexposed groups.

**Note:** An Explanation and Elaboration article discusses each checklist item and gives methodological background and published examples of transparent reporting. The STROBE checklist is best used in conjunction with this article (freely available on the Web sites of PLoS Medicine at http://www.plosmedicine.org/, Annals of Internal Medicine at http://www.annals.org/, and Epidemiology at http://www.epidem.com/). Information on the STROBE Initiative is available at www.strobe-statement.org.


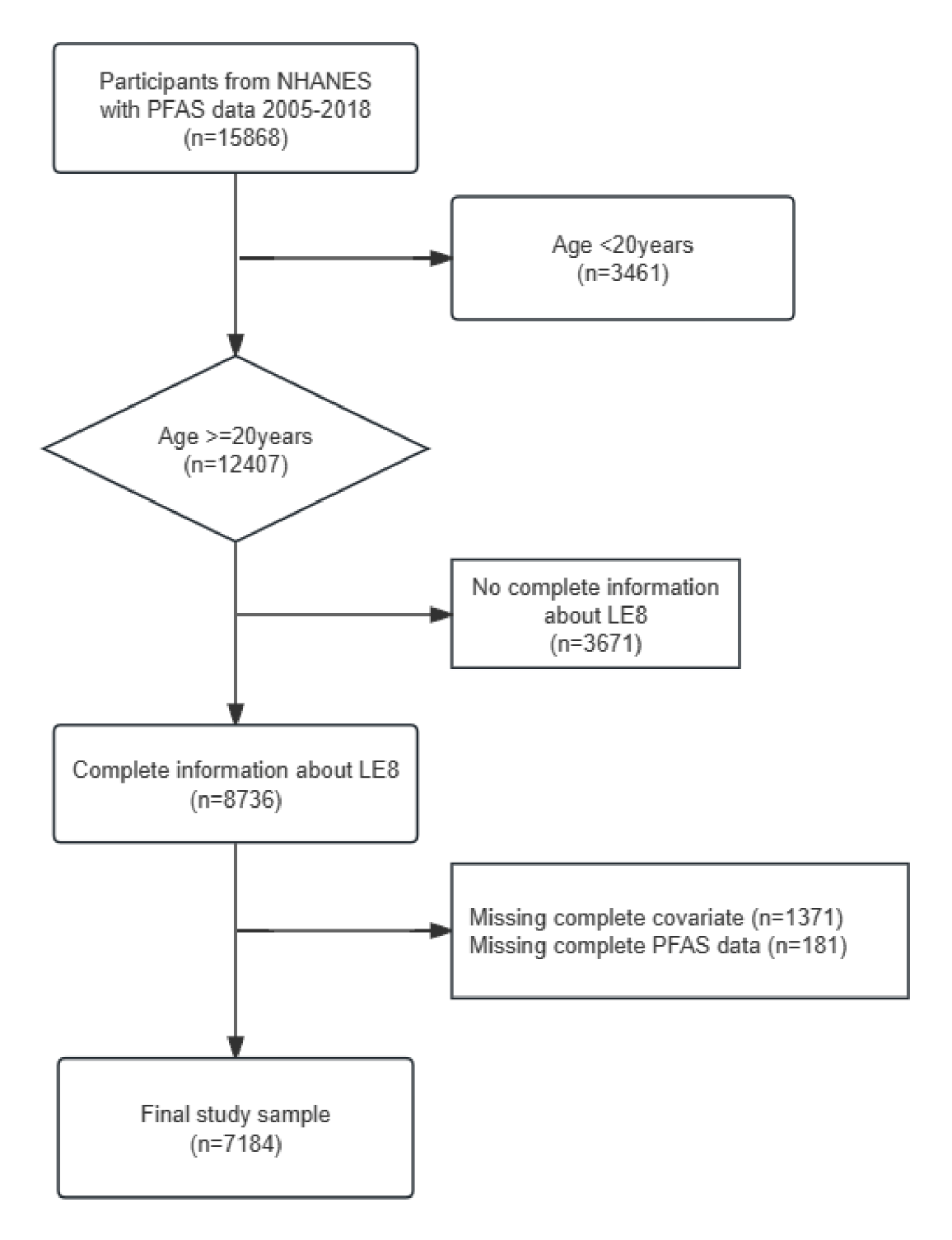


**Supplementary Figure 1.** Flow chart illustrating the inclusion and exclusion criteria for the study participants.


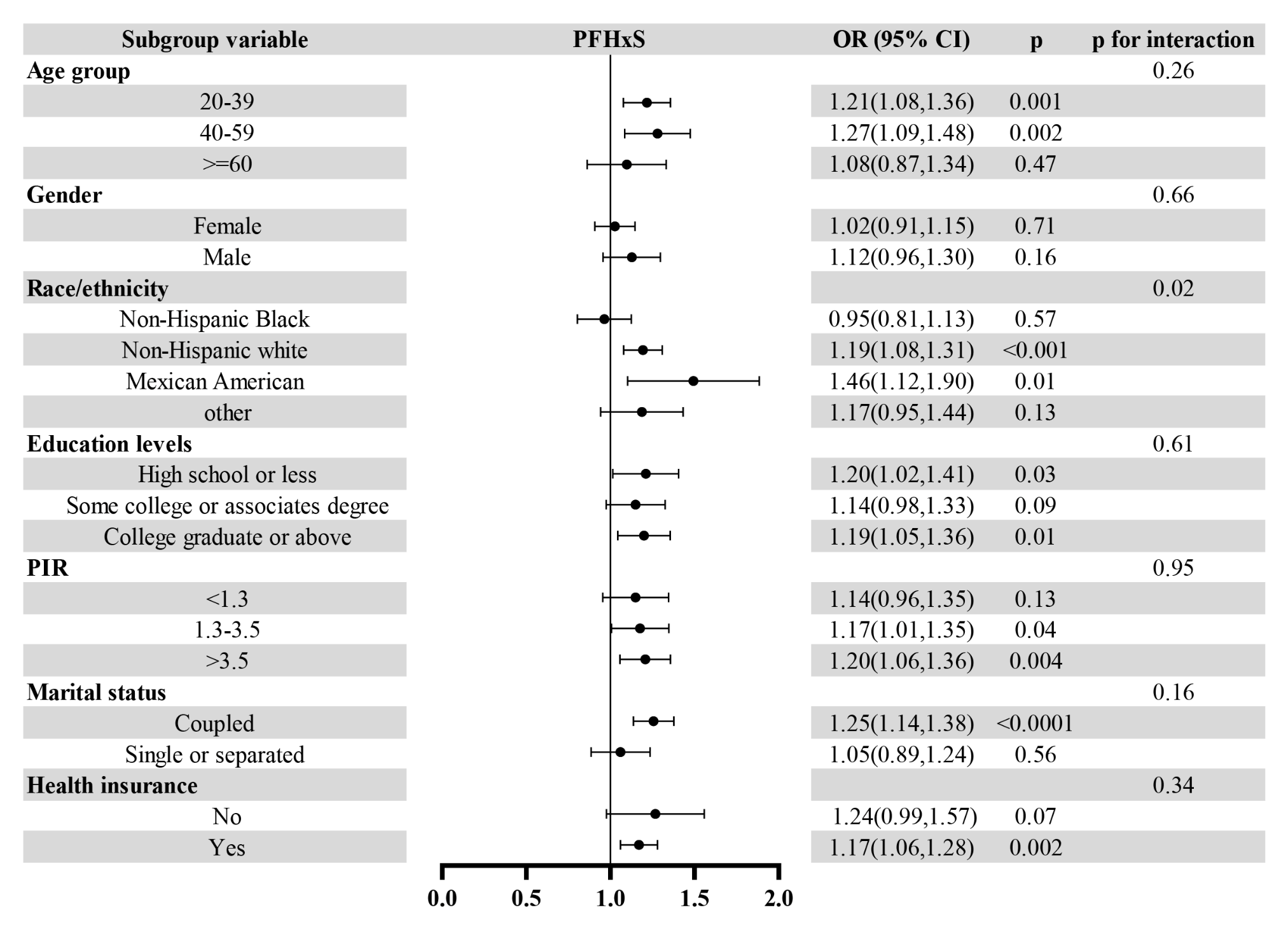


**Supplementary Figure 2.** Subgroup and interaction analyses of PFHxS and impaired CVH.


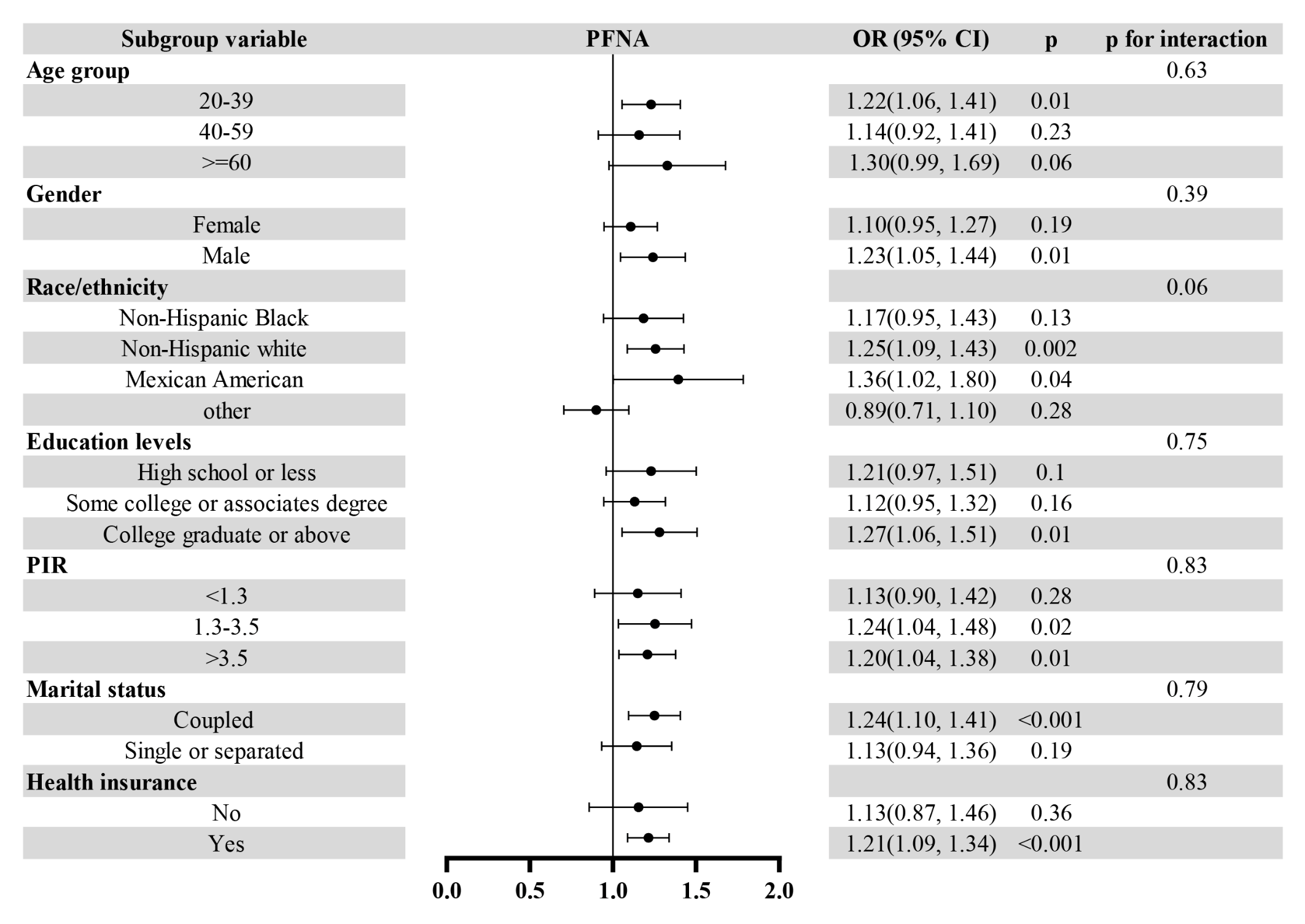


**Supplementary Figure 3.** Subgroup and interaction analyses of PFNA and impaired CVH.


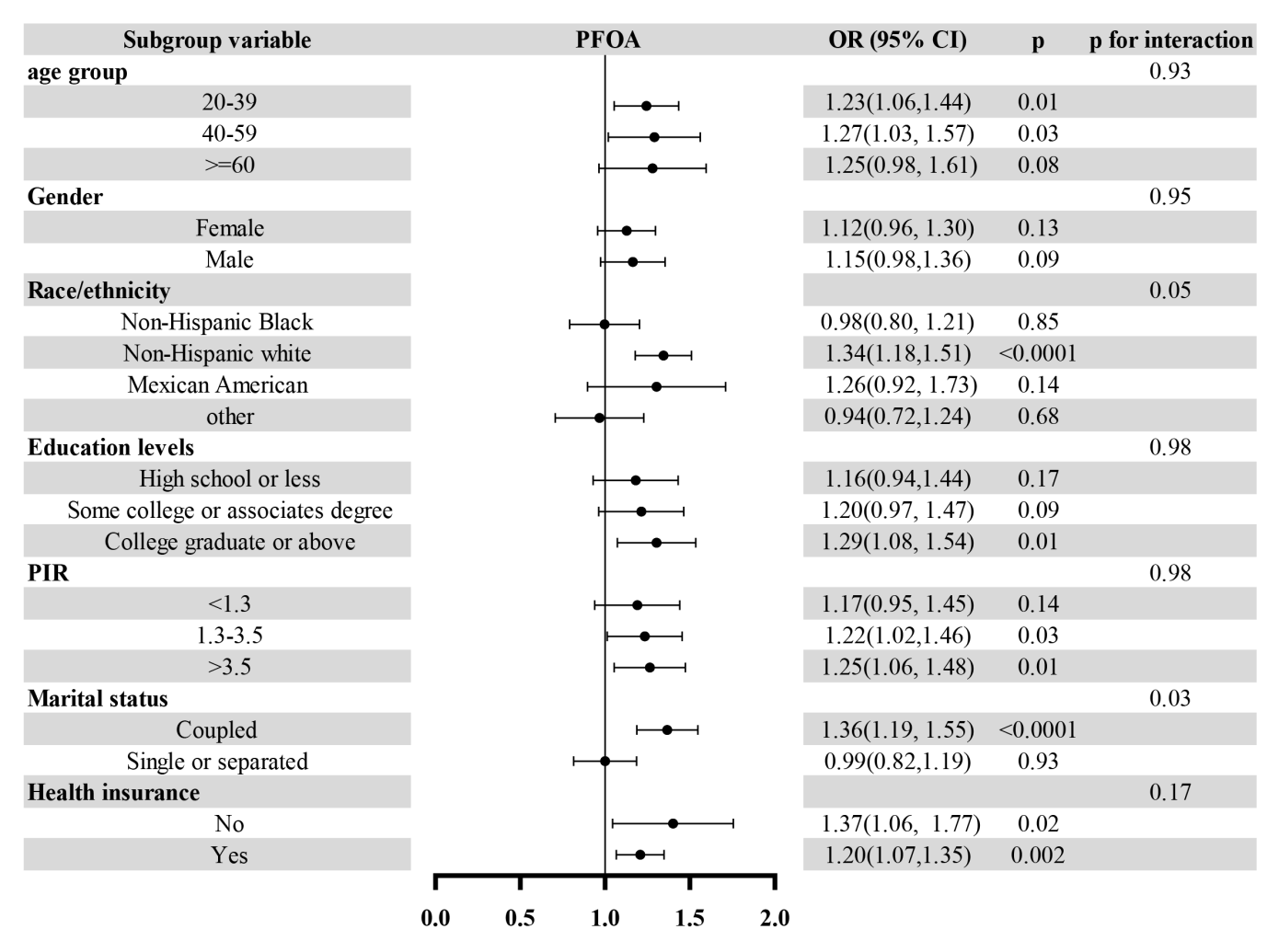


**Supplementary Figure 4.** Subgroup and interaction analyses of PFOA and impaired CVH.


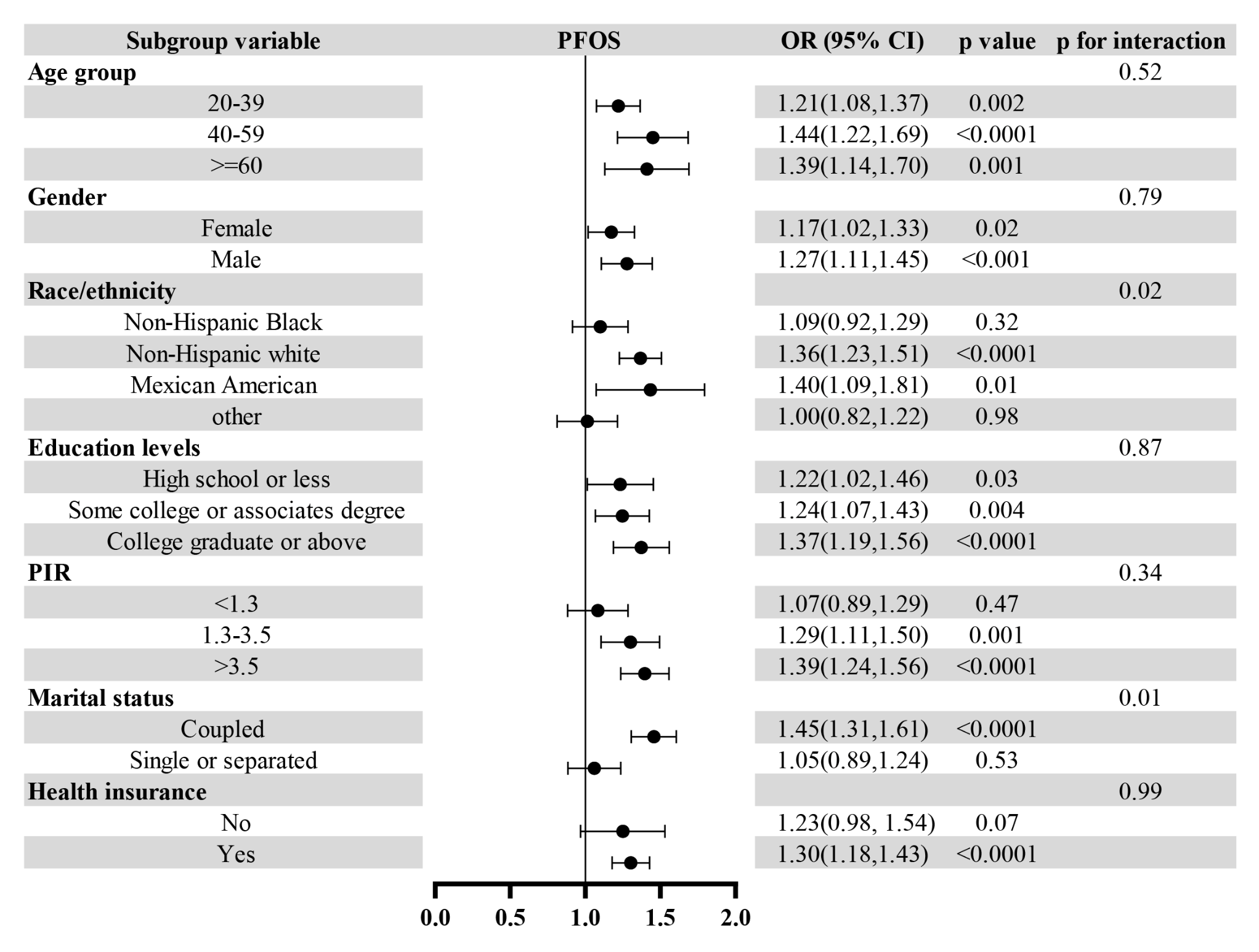
**Supplementary Figure 5.** Subgroup and interaction analyses of PFOS and impaired CVH.


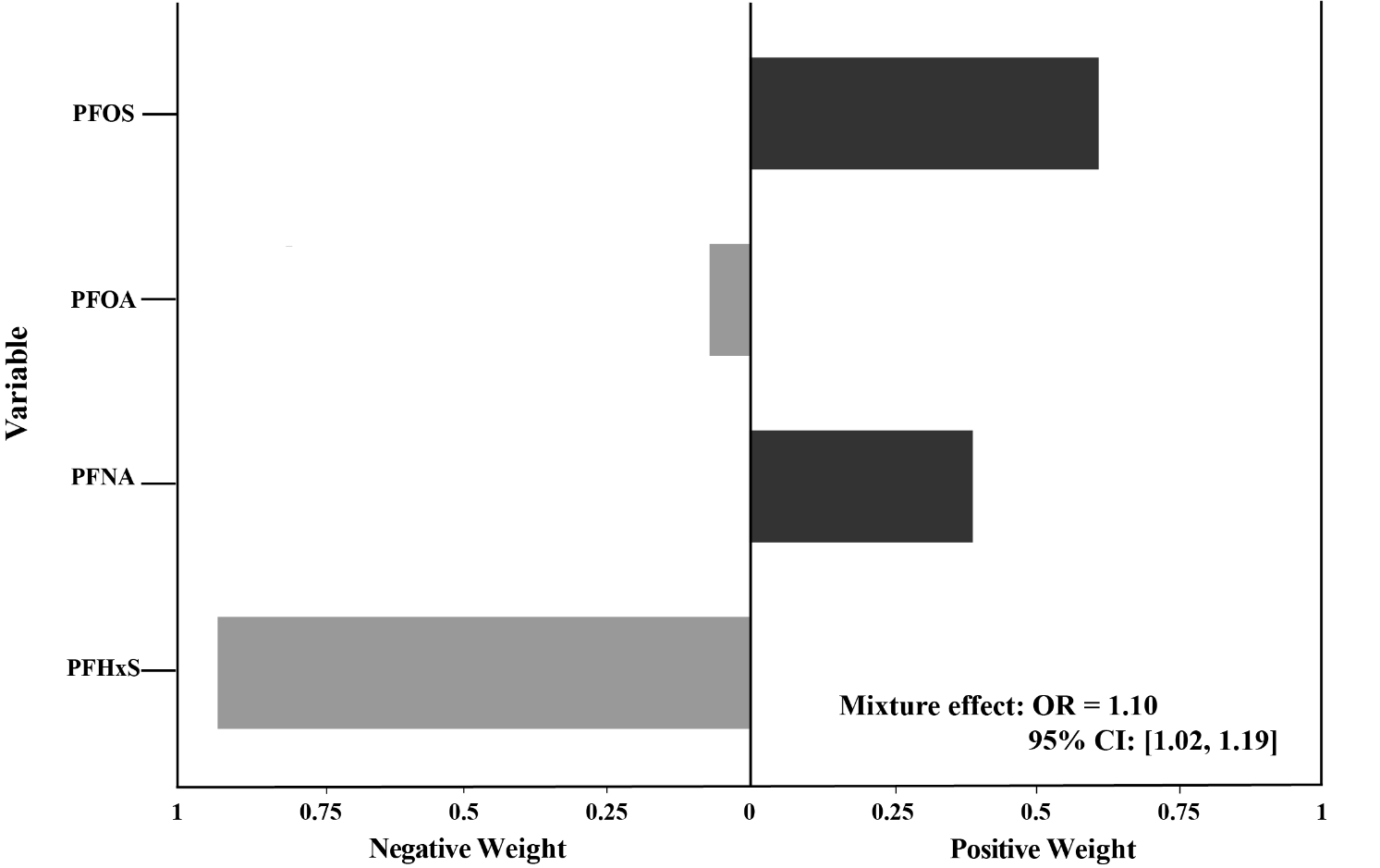


**Supplementary Figure 6:** Quantile g-computation scaled weights for each PFAS in the PFAS mixture.
